# Imputation Performance in Latin American Populations: Improving Rare Variants Representation With the Inclusion of Native American Genomes

**DOI:** 10.3389/fgene.2021.719791

**Published:** 2022-01-03

**Authors:** Andrés Jiménez-Kaufmann, Amanda Y. Chong, Adrián Cortés, Consuelo D. Quinto-Cortés, Selene L. Fernandez-Valverde, Leticia Ferreyra-Reyes, Luis Pablo Cruz-Hervert, Santiago G. Medina-Muñoz, Mashaal Sohail, María J. Palma-Martinez, Gudalupe Delgado-Sánchez, Norma Mongua-Rodríguez, Alexander J. Mentzer, Adrian V. S. Hill, Hortensia Moreno-Macías, Alicia Huerta-Chagoya, Carlos A. Aguilar-Salinas, Michael Torres, Hie Lim Kim, Namrata Kalsi, Stephan C. Schuster, Teresa Tusié-Luna, Diego Ortega Del-Vecchyo, Lourdes García-García, Andrés Moreno-Estrada

**Affiliations:** ^1^ Laboratorio Nacional de Genómica para la Biodiversidad (UGA-LANGEBIO), Unidad de Genómica Avanzada, Irapuato, Mexico; ^2^ Wellcome Trust Centre for Human Genetics, University of Oxford, Oxford, United Kingdom; ^3^ Instituto Nacional de Salud Pública, Cuernavaca, Mexico; ^4^ Centro de Ciencias Genómicas, Universidad Nacional Autónoma de México, Cuernavaca, Mexico; ^5^ Nuffield Department of Medicine, The Jenner Institute, University of Oxford, Oxford, United Kingdom; ^6^ Unidad de Biología Molecular y Medicina Genómica, Instituto Nacional de Ciencias Médicas y Nutrición Salvador Zubirán (INCMNSZ), Mexico City, Mexico; ^7^ Departamento de Economía, Universidad Autónoma Metropolitana, Mexico City, Mexico; ^8^ Departamento de Endocrinología y Metabolismo, Instituto Nacional de Ciencias Médicas y Nutrición Salvador Zubirán, Unidad de Investigación de Enfermedades Metabólicas, Mexico City, Mexico; ^9^ Tecnológico de Monterrey, Escuela de Medicina y Ciencias de la Salud, Monterrey, Mexico; ^10^Singapore Centre on Environmental Life Sciences Engineering, Nanyang Technological University, Singapore; ^11^GenomeAsia 100K (GA100K) Consortium, Singapore; ^12^ School of Biological Science, Nanyang Technological University, Singapore; ^13^ Instituto de Investigaciones Biomédicas de la UNAM, Mexico City, Mexico; ^14^ Laboratorio Internacional de Investigación sobre el Genoma Humano (LIIGH), UNAM, Juriquilla, Mexico

**Keywords:** Imputation, reference panels, GWAS, Native American ancestry, Latin Americans, underrepresented populations

## Abstract

Current Genome-Wide Association Studies (GWAS) rely on genotype imputation to increase statistical power, improve fine-mapping of association signals, and facilitate meta-analyses. Due to the complex demographic history of Latin America and the lack of balanced representation of Native American genomes in current imputation panels, the discovery of locally relevant disease variants is likely to be missed, limiting the scope and impact of biomedical research in these populations. Therefore, the necessity of better diversity representation in genomic databases is a scientific imperative. Here, we expand the 1,000 Genomes reference panel (1KGP) with 134 Native American genomes (1KGP + NAT) to assess imputation performance in Latin American individuals of mixed ancestry. Our panel increased the number of SNPs above the GWAS quality threshold, thus improving statistical power for association studies in the region. It also increased imputation accuracy, particularly in low-frequency variants segregating in Native American ancestry tracts. The improvement is subtle but consistent across countries and proportional to the number of genomes added from local source populations. To project the potential improvement with a higher number of reference genomes, we performed simulations and found that at least 3,000 Native American genomes are needed to equal the imputation performance of variants in European ancestry tracts. This reflects the concerning imbalance of diversity in current references and highlights the contribution of our work to reducing it while complementing efforts to improve global equity in genomic research.

## Introduction

Over the past years, GWAS have identified thousands of genetic associations to multiple phenotypes (MacArthur et al., 2017; Visscher et al., 2017), targets for potential new drugs (Agrawal and Brown 2014; Flannick et al., 2014; Nelson et al., 2015), and facilitated disease stratification (Chatterjee, Shi, and García-Closas 2016). However, most GWAS have been performed in populations with European ancestry (Popejoy and Fullerton 2016). Unfortunately, the findings of large-scale GWAS performed in populations of European descent have limited portability to other ancestry groups (Duncan et al., 2019; Sirugo, Williams, and Tishkoff 2019) due to population substructure. This represents a major limitation in the case of Latin American populations as they are the result of recent admixture primarily between Native American, European, and African populations, and only 1.3% of both discovery and replication studies have been performed in these populations (Mills and Rahal 2019). Furthermore, the genetic composition of Latin American populations is heterogeneous between countries (Chacón-Duque et al., 2018; Soares-Souza et al., 2018) and within countries (Moreno-Estrada et al., 2014; Harris et al., 2018; Kehdy et al., 2015). Different demographic histories often lead to different associated variants to a given phenotype (Martin et al., 2017). For example, variants in the *SLC16A11* gene have been associated with an increased risk of diabetes in Mexicans and appear to be segregating at low frequency in Latin American populations specifically (SIGMA Type 2 Diabetes Consortium et al., 2014). Likewise, risk variants of renal disease in *APOL1* associated with renal disease in west African populations are also found in the Americas as a result of the Transatlantic slave trade, differentially shaping the frequency spectrum of disease variants among Afro-descendent Latino populations (Nadkarni et al., 2018). If the current bias in catalogs of human variation persists, many population-specific variants will be overlooked, and precision medicine strategies will not benefit all populations equally (Martin et al., 2019).

A critical step when performing a GWAS is genotype imputation, which leverages linkage disequilibrium (LD) structure and haplotype sharing to estimate untyped variation in a SNP array based on a reference panel (Marchini et al., 2007). Genotype imputation increases statistical power, improves fine-mapping of association signals, and facilitates meta-analysis (Marchini and Howie 2010). Currently, available imputation panels do not have an explicit representation of Native American genomes. A previous study showed that in Latin American populations, SNPs in chromosomal segments with Native American ancestry have reduced imputation quality compared to those in chromosomal segments of European ancestry (Martin et al., 2017). Therefore, association signals coming from chromosomal segments with Native American ancestry will be harder to detect. This limits the scope and impact of biomedical research in the region.

Several projects and initiatives around the world are contributing to revert this trend (GenomeAsia100K Consortium 2019; Mulder et al., 2018; Gurdasani et al., 2015; Magalhães et al., 2018). For example, the Ugandan Genome Resource (Gurdasani et al., 2019) comprises genome-wide data for 6,400 individuals, including a subset of 1,978 whole genomes, which is enabling researchers to explore the genetic substructure of the region, improve imputation in African populations, and foster the discovery of novel association signals. In Latin America, recent sequencing efforts have generated whole-genome data from dozens of Native American genomes, including the Peruvian Genome Project (Harris et al., 2018) and the 12G and 100G-MX Projects (Romero-Hidalgo et al., 2017; Aguilar-Ordoñez et al., 2021) from the National Institute of Genomic Medicine (INMEGEN) in Mexico. However, only a subset of the existing generated data is available to the scientific community given the data sharing mechanisms implemented in each country. An ongoing multi-institutional effort in Mexico, the MX Biobank Project, is generating genome-wide data for more than 6,000 individuals nationwide, including 50 whole genomes of Native American ancestry representing the genetic variation of indigenous diversity within Mexico (http://www.mxbiobankproject.org). At a global scale, the inclusion of diverse populations in disease association research has been well demonstrated by the PAGE study (Wojcik et al., 2019), which combines genome-wide data for 49,839 individuals with diverse ancestries, enabling the discovery of novel association signals to well-studied phenotypes. Here, we combine novel and publicly available data from multiple sources to build a population-specific reference panel of Native American variation aimed at improving imputation performance in Latin American populations by expanding the current and widely used reference of the 1,000 Genomes Project (1KGP) (The 1000 Genomes Project Consortium et al., 2015) with 134 Native American genomes. Using a demographic simulation framework, we also explore the number of additional reference genomes that should be sequenced to bridge the gap in imputation quality between different ancestries. Strengthening these efforts in diverse populations is not only a question of equality in genomics, but it also entails the scientific advantage of furthering our understanding of complex phenotypes in biomedical research.

## Materials and Methods

### Building a Native American Reference Panel

**FIGURE 1:**
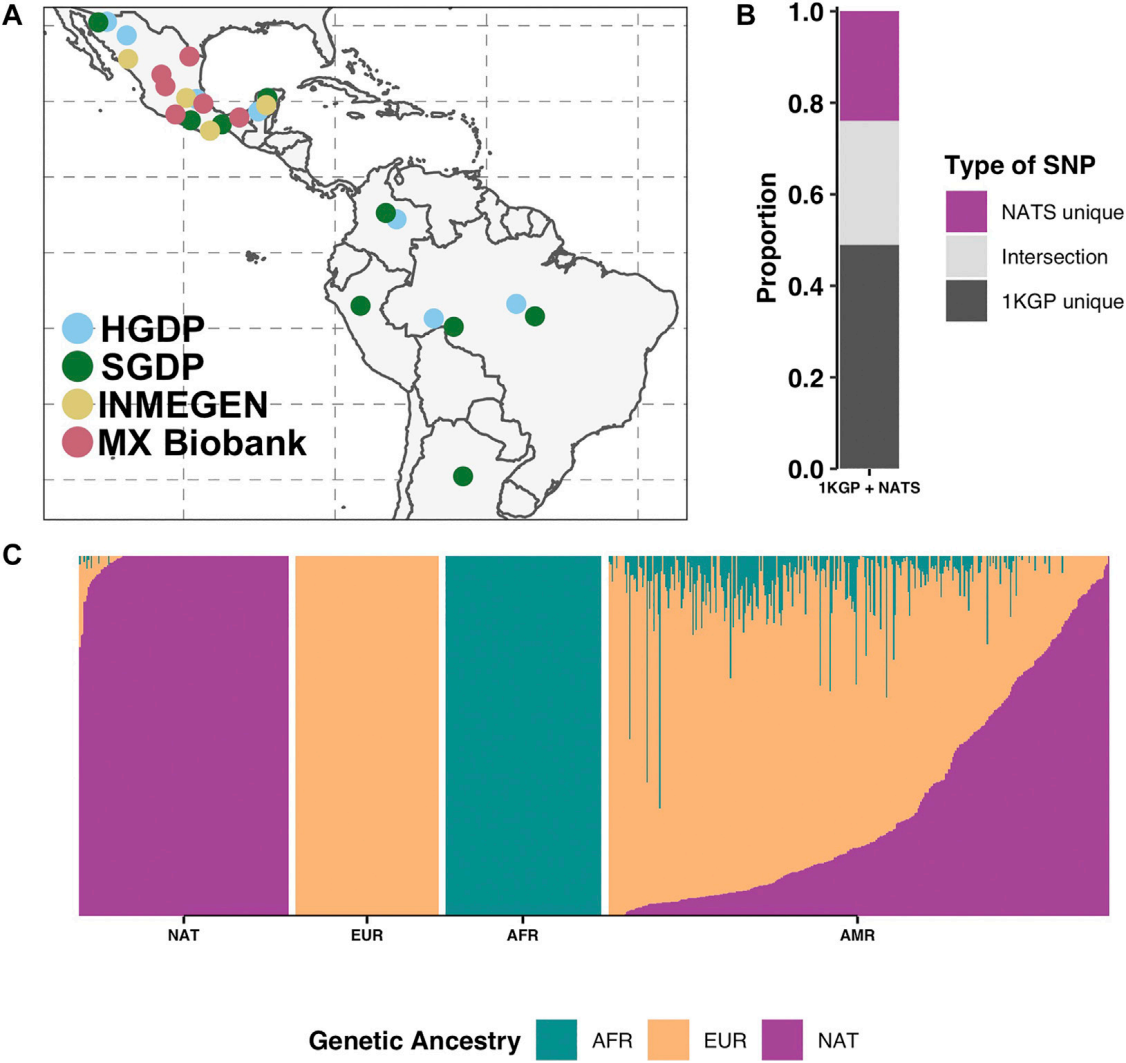
Native American reference panel (NATS). **(A)** Geographical sampling locations of the NATS reference panel. Colors represent the four data sources: HGDP (61) (Bergström et al., 2020), SGDP (11) (Mallick et al., 2016), INMEGEN (12) (Romero-Hidalgo et al., 2017), and MX Biobank (50) totaling 134 genomes. **(B)** SNP proportions of the union of 1KGP and NATS (1KGP + NATS) by SNP sharing categories. We show the proportion of SNPs unique to 1KGP, SNPs unique to the NATS panel, and the intersection. **(C)** Unsupervised ADMIXTURE analysis at *K* = 3 of the NATS reference panel (far left, *N* = 134) together with 104 European (CEU), 113 African (YRI), and 347 admixed Latin American (AMR) samples from 1KGP. Genetic ancestry abbreviations: AFR—African, EUR—European, NAT—Native American.

Our panel consists of 134 Native American individuals broadly distributed across the continent (Figure 1; Supplementary Tables S1, S2). We gathered publicly available whole-genome sequencing (WGS) data from HGDP (Bergström et al., 2020) (61 individuals), SGDP (Mallick et al., 2016) (11 individuals), and INMEGEN (Romero-Hidalgo et al., 2017) (12 individuals). Additionally, we whole-genome sequenced the genome of 50 Mexican individuals with the highest Native American ancestry (99% on average) from the MX Biobank Project (http://www.mxbiobankproject.org). These were selected to maximize indigenous ancestry and geographical representation across Mexico. Individual genetic ancestry proportions were estimated using ADMIXTURE (Alexander, Novembre, and Lange 2009) at *K* = 3 using Utah residents with Northern and Western European ancestry (CEU), Yoruba in Ibadan, Nigeria (YRI), and the Latin Americans (AMR) of 1KGP as references.

To construct the panel, we restricted the datasets to biallelic SNPs with no missing data in any individual across each data source. This was done for all four data sources (Supplementary Table S3). The data processing was done using *VCFtools v0.1.17* (Danecek et al., 2011). Then, we merged the data using *bcftools v1.9* (Danecek et al., 2021) using the flag --missing-to-ref that fills the missing positions in one panel but present in another with homozygous reference. To minimize any potential bias introduced with this strategy, we made sure that any previously removed position in any of the sources was not present in the final freeze. The final dataset consists of a total of 10,981,451 SNPs.

Finally, we phased the data using *SHAPEIT2 v2. r837* (Delaneau et al., 2014) using the following flags: --window 0.5 --states 500 --burn 10 --prune 10 --main 50. Then, we converted the data to the reference format used by *IMPUTE2* (Howie et al., 2012). We named this panel NATS.

### Whole-Genome Sequencing and Variant Calling

Fifty individuals from the MX Biobank Project were sequenced at 40X on Illumina HiSeqX instruments using dual indexed barcodes. The raw reads were aligned to the human genome assembly GRCh37 using *BWA v.0.7.17-r1198-dirty* (Li and Durbin 2009). We added the mate tags with *Samblaster v0.1.24* (Faust and Hall 2014) and used *Sambamba v0.7.1* (Tarasov et al., 2015) for file conversion and sorting. To generate the alignment statistics, we used *Samtools v1.10* (Li 2011) with the option *depth -a*. Finally, we performed variant calling and generated the final gvcf files with *GATK v4.1.9.0* (McKenna et al., 2010) using the human genome assembly GRCh37 as the reference genome. Details are available as part of the Supplementary Material (Supplementary Table S2; Supplementary Figure S9).

### Creating a SNP Array Subset From WGS Data for Imputation Performance Evaluation

To evaluate the performance of our panel, we used WGS data from the 347 AMR individuals in 1KGP as target individuals for imputation. Namely, Puerto Ricans in Puerto Rico (PUR), Peruvians in Lima (PEL), Colombian in Medellin (CLM), and Mexican ancestry in Los Angeles (MXL). We generated an array dataset by subsetting the AMR individual genomes to the existing positions in the Multi-Ethnic Global Array (MEGA) using *VCFtools v.0.1.17* and saved the removed positions from the WGS data to use for imputation validation. Illumina’s MEGA array includes nearly 1.8 M markers (1,779,819) genome-wide distributed and was designed to leverage SNP content from various global sequencing efforts, mostly Phase 3 of the 1,000 Genomes Project. To better approximate a real scenario, we unphased the array dataset with *Plink v1.9* (Chang et al., 2015) by transforming the data to bed format. Finally, we phased the dataset again with *SHAPEIT2 v2. r837* using 1KGP as a phasing reference.

### Local Ancestry Inference

To evaluate the performance by ancestry, we deconvoluted local ancestry for the Latin American individuals from 1,000 Genomes. We used 70 YRI individuals in 1KGP as the African reference, 70 CEU individuals from 1KGP as the European reference, and 70 Native American individuals from (Moreno-Estrada et al., 2014) as the Native American reference. The selected individuals had the highest African, European, and Native American genetic components, respectively. We used the PopPhased version of *RFMix v.1.5.4* (Maples et al., 2013) with the following flags: -w 0.2 -e 0 --forward-backward.

### Imputation and Imputation Performance

We implemented a leave-one-out strategy for imputation. Namely, the target individual was removed from the 1KGP reference. We performed imputation with *IMPUTE2* for chromosomes 2 and 9. These chromosomes, being the largest and of intermediate size, respectively, were selected to ensure a representative subset of variants across the genome while keeping the project within the available computational capacity. We used 1KGP and 1KGP + NATS as reference panels. When using 1KGP as a reference, we used the flag --k_haps 1,000, and when using 1KGP + NATS, we used the flags --merge-ref-panels and --k_haps 1,250.

We obtained the imputed dosages with the formula: P(Aa) + 2P(aa). We computed the Pearson squared correlation (*r* ^2^) between the imputed dosages and the real dosages for each individual using R software. Overall imputation accuracy was stratified by minor allele frequency and local ancestry diplotype (AFR_AFR, AFR_EUR, AFR_NAT, EUR_EUR, EUR_NAT, NAT_NAT). We also compared the number of SNPs above the GWAS quality threshold (MAF >=0.01 and INFO >0.3) for both reference panels stratified by local ancestry diplotype in the target individuals.

### Demographic Simulation

We simulated neutral genetic sequence data under a coalescent model. We used the *msprime* (Kelleher, Etheridge, and McVean 2016) option of *stdpopsim* (Adrion et al., 2020) to simulate a previously defined American admixture model for Latinos (Browning et al., 2018). It models African, European, and Asian (as Native American proxy) demographic history and an admixture event taking place 12 generations ago. In the absence of realistic admixture models that use Native American instead of East Asian genomes as proxy in the simulations and based on the framework described by Browning et al. (2018), we will now refer to the simulated Asian population as Native American for the purpose of predicting imputation performance at incremental numbers of reference genomes in a similar scenario to the Latin American admixture. The simulated admixed population ancestral proportions are 1/6 African, ⅓ European, and ½ Native American. In total, we simulated chromosome 9 for 661 Africans, 503 Europeans, 3,000 Native Americans, and 657 admixed individuals. We selected all the Africans, Europeans, and the first 347 admixed individuals to serve as the base reference panel (note that these numbers mirror the sample sizes of 1KGP for each ancestry). The remaining 300 admixed individuals were used as imputation targets, and incremental subsets of the 3,000 Native American genomes were added sequentially to the base reference panel.

To simulate genotype array data for the target individuals, we downsampled the simulated neutral sequence to match the allele frequency spectrum in European populations of 1KGP and the average distance between SNPs of the MEGA array. We used the European populations in 1KGP to mirror the ascertainment bias towards European ancestry in current array designs. We estimated local ancestry using *RFMix* for the 300 admixed individuals used as imputation targets. We randomly selected 100 simulated individuals from each ancestral population (African, European, and Asian) as references for the local ancestry inference. Here, again, we used Asians as the closest proxy for Native Americans in the available simulation model.

We conducted imputation with the base reference panel plus a varying number of additional reference genomes (0, 100, 134, 200, 400, 600, 800, 1,000, 1,500, 2000, and 3,000). Finally, we compared imputation *r* ^2^ of using different reference panels stratified by local ancestry and allele frequency in the target individual genomes.

## Results

### The Native American Reference Panel NATS

We built a Native American reference panel (NATS) representing indigenous populations across Latin America. The panel consists of publicly available data [HGDP (Bergström et al., 2020), SGDP (Mallick et al., 2016), and INMEGEN (Romero-Hidalgo et al., 2017)] and 50 new genomes from the MX Biobank Project (*Materials and Methods*, and Supplementary Table S2). While most of the genomes in the panel are from indigenous groups in Mexico (103 of 134; 76.8%) (Figure 1A; Supplementary Table S1), our panel also encompasses native groups from Colombia, Brazil, and Peru. When merging NATS with 1KGP, the total number of SNPs is 102,336,497, of which 24,518,242 (24%) are unique to our panel (Figure 1B). The amount of non-indigenous admixture in our panel is less than 1.5% overall (Figure 1C). Only some Mayan individuals from HGDP show between 0.8 and 23% of European admixture (on average 6%) (Supplementary Table S1). Overall, our panel has 98.5% of Native American genetic ancestry. We acknowledge that, while this panel includes as many genomes as possible from those publicly available at the time of publication, it does not fully capture the genetic variation of the vast ethnic diversity in the continent. It is intended to serve as a first approximation to evaluate the impact of ancestry representation in imputation performance.

### Imputation Performance of the NATS Reference Panel

To assess the impact of our panel on imputation performance, we imputed the AMR individuals (from Colombia, Peru, Puerto Rico, and Mexico in 1KGP) at SNPs not found on the MEGA array using a leave-one-out strategy, with either 1KGP or 1KGP + NATS as reference panels (Materials and Methods). We chose the MEGA array because it was specifically designed to capture global variation better. We compared the mean number of SNPs above the standard quality threshold for human genetic studies (MAF >= 1% and INFO >= 0.3) using the two reference panels. We were able to increase the number of SNPs above the quality threshold across the four populations using our NATS panel (Table 1). The magnitude of the increase is correlated with the individual’s proportion of native ancestry (Supplementary Figure S1). Furthermore, the majority of these SNPs fall into diploid European tracts of the genome (Supplementary Figure S2) regardless of the ancestry composition of each population, and which reference panel was used for imputation. This is because even though the 1KGP has as many African individuals as Europeans, European ancestry is more predominant in AMR individuals.

**TABLE 1:** SNPs above the standard quality threshold using both panels after imputing missing variants. We show the average number of SNPs with MAF >= 0.01 and INFO >= 0.3 using both reference panels and the overall proportion of Native American ancestry of the population. *p*-value was calculated with a two-tailed paired *t*-test. The average number of SNPs with MAF <0.01 and INFO >0.3 for both panels is shown in Supplementary Table S4.

Population	SNPs above quality threshold (1KGP)	SNPs above quality threshold (1KGP + NATS)	Increase of SNPs using 1KGP + NATS	Average proportion of Nat. American ancestry
Peru (PEL)	244,818	248,087	3,269 (*p*-value = 2.03e-49)	0.70
Mexico (MXL)	265,619	268,254	2,635 (*p*-value = 6.5e-31)	0.42
Colombia (CLM)	279,828	281,911	2,163 (*p*-value = 8.3e-47)	0.18
Puerto Rico (PUR)	291,035	292,734	1,699 (*p*-value = 2.9e-67)	0.06

**FIGURE 2:**
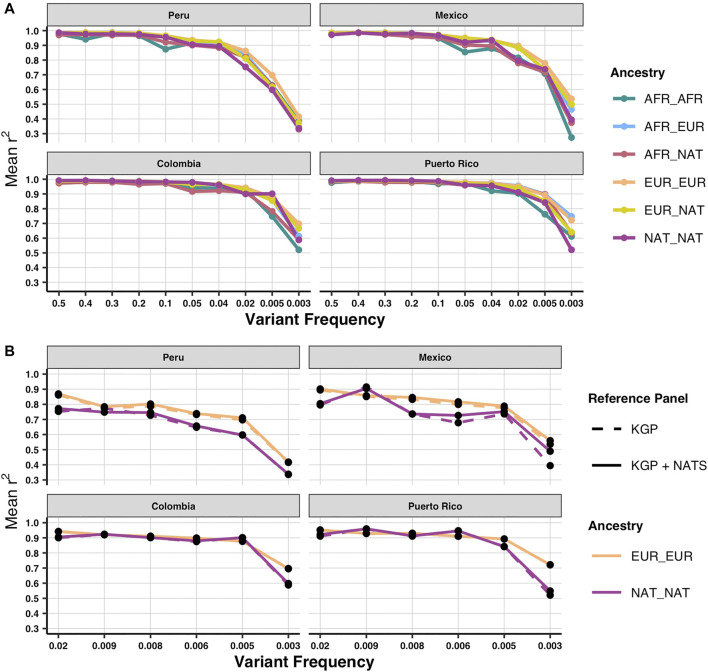
Imputation accuracy by local ancestry and population using both reference panels. **(A)** Imputation accuracy of the four AMR populations stratified by diploid local ancestry for the MEGA array using 1KGP as reference panel. **(B)** Imputation accuracy for the Native and European diploid ancestries using 1KGP and 1KGP + NATS as reference panel focusing on rare alleles. Imputation accuracy was calculated with the Pearson squared correlation between imputed and real allele dosages.

To determine imputation accuracy, we computed the correlation between the real allele dosages and the imputed dosages (*Materials and Methods*). We checked imputation accuracy in 1KGP admixed individuals trimmed down to SNP array positions stratified by diploid ancestry (Figure 2A). Overall, imputation accuracy is worse in AMR populations with the highest proportion of Native American ancestry (Supplementary Figure S3). As previously reported (Martin et al., 2017), the ancestry tracts that perform the worst are the ones that are underrepresented in the reference panel, specifically African and Native American. Next, we evaluated imputation accuracy using our panel (1KGP + NATS). We were able to increase imputation accuracy particularly in rare alleles (frequency >0.003 and <0.008) with diploid Native ancestry of the Mexican population (*p*-value < 0.05 two-tailed paired *t*-test) (Figure 2B) but not for the other populations (Supplementary Figure S3) or in common frequencies (Supplementary Figure S4). Interestingly, we do not see the same increase in the Peruvian population, which has the highest proportion of Native American ancestry overall. This could be explained by the fact that the majority of our reference data comes from native Mexicans (Figure 1A; Supplementary Table S1). Since rare variants tend to be more private to each population (Biddanda, Rice, and Novembre 2020), we could better impute rare alleles in admixed Mexicans. This suggests that, to see a similar improvement in accuracy in the other populations, we would need to include more native individuals from each local region. Surprisingly, we could also see an improvement in diploid European ancestry tracts in the Mexican population (*p*-value < 0.05 two-tailed paired *t*-test for SNPs with frequency >0.003) (Figure 2B). One possible explanation is that because our NATS reference panel still keeps a minor fraction of European ancestry, some European haplotypes at higher frequency in Mexico could be better captured by reference genomes with such a genetic mixture. In some cases, like variants of frequency <0.02 and >0.009 with diploid Native ancestry in PEL, we could also observe a slight decrease in imputation accuracy using NATS. This could result from the uncertainty added to the data in the cross-imputation step that *IMPUTE2* performs when merging two reference panels (Howie, Marchini, and Stephens 2011).

### Predicting Imputation Improvement From Additional Native American Genomes Using Simulations

Our results show that after adding 134 Native American genomes to the most widely used reference panel of global variation, we observe a promising trend of improvement. Still, we do not come close enough to equal the imputation performance for other better represented ancestries. The question remains of how many additional genomes are still needed to close the gap. To explore this, we employed demographic simulations using *stdpopsim* (Adrion et al., 2020) and *msprime* (Kelleher, Etheridge, and McVean 2016) to generate data for a previously defined American admixture model (Browning et al., 2018). This approach allows us to explore a simulated scenario where three divergent populations intermingle to form a new admixed population (like it occurred in Latin America). With this, we can replicate the current situation where reference data are mostly available for two of the three source populations. By being able to simulate any amount of data, we can assess how many genomes of the underrepresented population (in our case, Native Americans) are necessary to equal imputation performance across ancestries. Briefly, the model simulates African, European, and Asian source populations. In the context of this analysis, the Asian population serves as a proxy for a Native American reference. We do not directly simulate a Native American population due to the lack of realistic admixture models that incorporate Native American instead of East Asian genomes as proxy in the inference of demographic parameters, which are needed to properly run the simulations. Building such demographic model is beyond the scope of this study, so given the available model and since this project focuses on Latin American populations, we will refer to the simulated Asian population as Native American. The model also simulates an admixed population that consists of 1/6 African, ⅓ European, and ½ Native American. We generated a base reference panel consisting of 661 Africans, 503 Europeans, and 347 admixed individuals (matching 1KGP sample sizes for those ancestries), as well as 3,000 Native American individuals to add sequentially to the base reference, and 300 additional admixed individuals as imputation targets (*Materials and Methods*).

We confirmed the ancestry proportions of our simulated data using *ADMIXTURE* (Supplementary Figure S5). To replicate the imputation pipeline, we created a genotype array dataset for the simulated target individuals by matching mean distance between markers and frequency in the European population of SNPs in the MEGA array to the simulated array, to mirror the bias in standard arrays (*Materials and Methods* and Supplementary Figure S6). Then, we imputed the 300 target individuals with the base reference plus either 0, 100, 134 (to mirror the sample size in NATS), 200, 400, 600, 800, 1,000, 1,500, 2000, or 3,000 Native Americans. We were able to recover roughly the same pattern of imputation accuracy (Supplementary Figure S7). Namely, accuracy decreased the less represented the ancestry was in the base reference with the Native American as the worst-performing ancestry. One caveat is that the best-performing ancestry is African contrary to what we see in the real data (Figure 2A). This is likely because the 661 African individuals are from the population that contributed to the admixed population in the simulation, which is not the case for real data. Different African ancestries contributed more or less to different Latin American populations (Micheletti et al., 2020) and not all are present in 1KGP.

**FIGURE 3:**
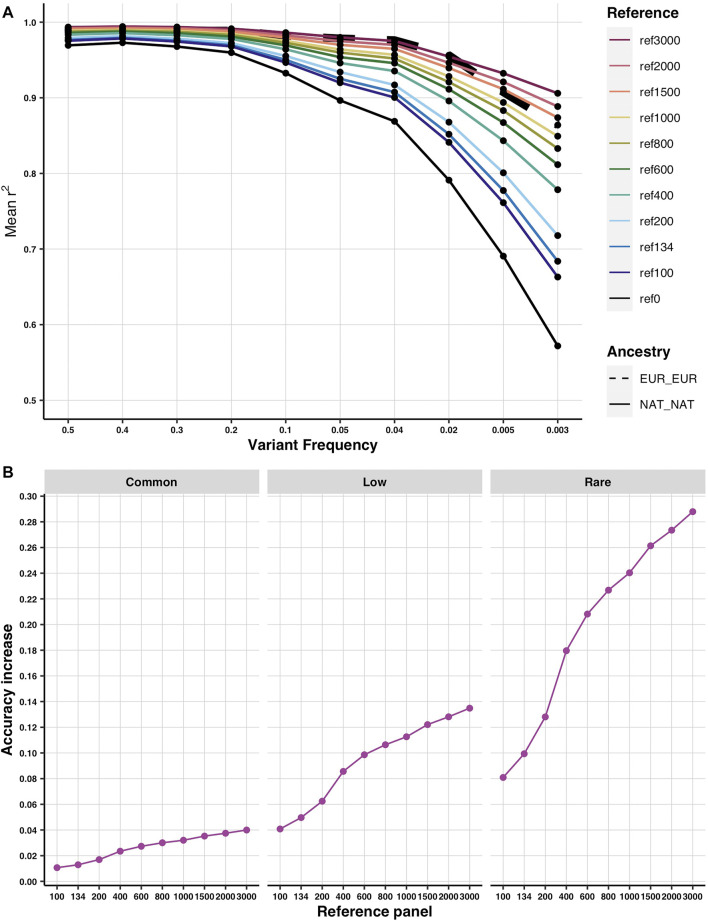
Predicted imputation accuracy according to demographic simulations. **(A)** Imputation accuracy in the diploid Native American (solid colored lines) and diploid European (thick dashed line) ancestries using different simulated reference panels of incremental sizes. Ref 0 stands for the base reference (as it has 0 additional reference genomes). Given the available demographic model (Browning et al., 2018), a simulated Asian population was used as a proxy for Native American ancestry for the purpose of reproducing a three-way admixture process with similar ancestry proportions of African, European, and Native American sources to that observed in admixed Latino populations (see Methods for details). **(B)** Increase in imputation accuracy from the base reference in the Native American diploid ancestry at increasing sizes of the reference panel by allele frequency [common (0.5–0.05), low (0.05–0.01), and rare (0.01–0.003)].

When incorporating additional Native American genomes, imputation accuracy only increased in those tracts with any Native ancestry (Supplementary Figure S8). Furthermore, for imputation accuracy in Native American diploid ancestry tracts to equal that in European diploid ancestry tracts, 3,000 Native genomes were needed for variants with frequency>=2%, while 1,500 were enough for variants with frequency <2% (Figure 3A). To ask whether we reach a saturation point in the increase of imputation accuracy in the Native diploid ancestry, we compared the difference between accuracy in the base reference versus each additional reference. As expected, the behavior is different for common (frequency >0.05), low (frequency <0.05 and >0.01), and rare (frequency <0.01) variants (Figure 3B). Neither of them seems to show a saturation point at 3,000 newly added Native genomes. The steepest increase is achieved for the rare alleles, whereas for the common alleles, the increase is slower. This agrees with the previous result where more genomes were needed to match the Native imputation accuracy to the European one for common variants. It is also evident that the variants of common frequency are closest to saturation in accuracy as their values were already close to one (Figure 3A).

## Discussion

GWAS requires large sample sizes to detect genetic associations to complex phenotypes, and more so as the field moves toward studying rare variants (Collins 2012; Amendola et al., 2018; Abul-Husn and Kenny 2019). Therefore, SNP array platforms will continue to inform GWAS even as whole-genome sequencing costs continue to drop. In this scenario, imputation tools and genome variation resources are vital to increasing the statistical power to discover associations in understudied populations. So far, GWAS have mainly focused on populations with European ancestry (Popejoy and Fullerton 2016; Mills and Rahal 2019) and, over the past years, interesting discoveries have been made (Visscher et al., 2017). However, not all GWAS results are portable between populations (Martin et al., 2017; Duncan et al., 2019; Sirugo, Williams, and Tishkoff 2019). To ensure that these advances reach all people equitably, we must expand these studies to other populations. Other recent projects around the world have sought to reverse this trend (Gurdasani et al., 2015, 2019; GenomeAsia100K Consortium 2019; Magalhães et al., 2018; Mulder et al., 2018) improving imputation accuracy, fine mapping of associations, and discovering novel associations to well-studied phenotypes. We sought to add to this trend by creating a Native American imputation reference panel merging publicly available Native American genomes (Mallick et al., 2016; Romero-Hidalgo et al., 2017; Bergström et al., 2020) with 50 novel genomes.

One major caveat of our panel is that it does not comprehensively reflect the indigenous genetic variation across the Americas. Most of the data come from individuals from Mexico. Furthermore, the 134 genomes added are only a small increment (5%) with respect to 1KGP. The contribution of this panel is small in comparison to projects like the Uganda Genome Resource that sequenced 1,978 novel genomes (Gurdasani et al., 2019). Even with these limitations in mind, we were able to quantify the consequences of the lack of Native American genomes in commonly used imputation reference panels using empirical and simulated data analyses, while highlighting what this means for ongoing and future studies in the region.

Our panel increased the number of SNPs above the standard quality threshold for human genetic studies increasing statistical power in the four AMR populations of 1KGP. This mirrors what has been achieved by other studies in other populations (Ahmad et al., 2017; Magalhães et al., 2018; Gurdasani et al., 2019). The magnitude of this increase is positively correlated with the proportion of Native American ancestry. In other words, our panel has a stronger impact on individuals with higher Native American ancestry. However, even after using our panel, the majority of SNPs that were above the quality threshold are in chromosomal segments of the genome with European diploid ancestry, regardless of the proportion of European ancestry in the population, due to an over-representation of this ancestry in the reference panel. This means that, when doing a GWAS, the genetic signals predominantly found on the European ancestry will be easier to detect.

We were able to increase imputation accuracy in rare variants of Native American diploid ancestry in the MXL population. This was not the case for the other three populations. We expected that, since PEL is the population with the highest Native American ancestry proportion, it would also be the population most benefited by the use of our extended panel. However, there can be high levels of genetic differentiation among Native American groups, even if they are geographically close (Moreno-Estrada et al., 2014). In light of this fact, it is not a surprise that our panel, constructed with a majority of Native American individuals from Mexico, only improves accuracy in the MXL population. This suggests that to observe similar results in other populations, we should include more individuals of those populations in our panel. We also observed an increase in accuracy in some variants of European diploid ancestry. This could be attributed to the small fraction of European admixture present in the whole genomes of our extended panel, despite being enriched for Native American ancestry. Also, some of these European haplotypes could have better-captured variation found in European ancestry segments of MXL individuals. Finally, to achieve an overall increase in imputation accuracy across the whole spectrum of variant frequencies as achieved in other studies (Ahmad et al., 2017; Gurdasani et al., 2019), we would need a larger Native American reference panel, as quantified by our simulations.

These results are important with regard to not only GWAS but also their further applications. For instance, one of the applications of GWAS summary statistics is Polygenic Risk Scores (PRS). PRS calculates the genetic “risk” of an individual for a particular phenotype by summing the risk alleles present in that individual (Torkamani, Wineinger, and Topol 2018). PRS necessitates summary statistics calculated in a population as close as possible to the target individuals to be accurate. Previous studies have shown that this is not a trivial task (Tropf et al., 2017; Sirugo, Williams, and Tishkoff 2019; Mostafavi et al., 2020). Even among European populations, PRS estimates vary widely depending on the source of summary statistics due to population structure (Berg et al., 2019; Sohail et al., 2019). To have accurate PRS for the Latin American population, we need to have more studies in the region. Furthermore, our results show that we also need a better imputation panel for these populations to avoid a bias towards identifying genetic signals present on the European ancestry background.

The question of how much data are needed remained. To answer it, we employed demographic simulations. We replicated the same pattern of imputation accuracy of our data and of previous studies (Martin et al., 2017). Our strategy shows that we would need at least 3,000 Native American genomes to equal imputation accuracy of Native diploid ancestry to that of European diploid ancestry across all variant frequencies. This number holds for populations such as MXL with roughly similar ancestral proportions as the simulated admixed population. The minimum number of necessary new genomes will change depending on the proportion of native ancestry of the target population. Our study provides a framework for future projects to decide how many resources to allocate to the generation of whole-genome data. Furthermore, we have shown that rare variants are the most benefited by the addition of new data. This will prove particularly relevant as the field moves towards studying that end of the variant frequency spectrum (Cirulli et al., 2020; Minikel et al., 2020). Overall, our results show the importance of generating more diverse imputation panels to enable genetic discoveries in a broader spectrum of human diversity and to procure equity as scientific advancements in precision medicine should extend globally in benefit of all.

## Data Availability

The newly generated data presented in the study are deposited in the European Genome-phenome Archive (EGA) repository, accession number EGAD00001008354 i.e. https://ega-archive.org/datasets/EGAD00001008354.
