# Supplementary material for: Imputation Performance in Latin American Populations: Improving Rare Variants Representation With the Inclusion of Native American Genomes
Source: Front Genet. 2022 Jan 3;12:719791. doi: 10.3389/fgene.2021.719791 (PMC8762266; doi:10.3389/fgene.2021.719791)
Supplement: Supplementary file 1 [file DataSheet1.PDF]

Supplementary Material

Imputation performance in Latin American populations: improving rare variants representation with the inclusion of Native American genomes

Andrés Jiménez-Kaufmann[1], Amanda Y. Chong[2], Adrián Cortés[2], Consuelo D. Quinto-Cortés[1], Selene Fernandez-Valverde[1], Leticia Ferreyra-Reyes[3], Luis Pablo Cruz-Hervert[3], Santiago G. Medina-Muñoz[1], Mashaal Sohail[1,4], Maria José Palma-Martinez[1], Gudalupe Delgado-Sánchez[3], Alexander J. Mentzer[2], Adrian V.S. Hill[2,5], Hortensia Moreno-Macías[6,7], Alicia Huerta-Chagoya[6], Carlos A. Aguilar-Salinas[8,9], Michael Torres[1], Hie Lim Kim[10,11,12], Namrata Kalsi[10], Stephan C. Schuster[10,11,12], María Teresa Tusié-Luna[6,13], Diego Ortega Del-Vecchyo[14], Lourdes García-García[3], Andrés Moreno-Estrada[1*].

1.1 Supplementary Figures

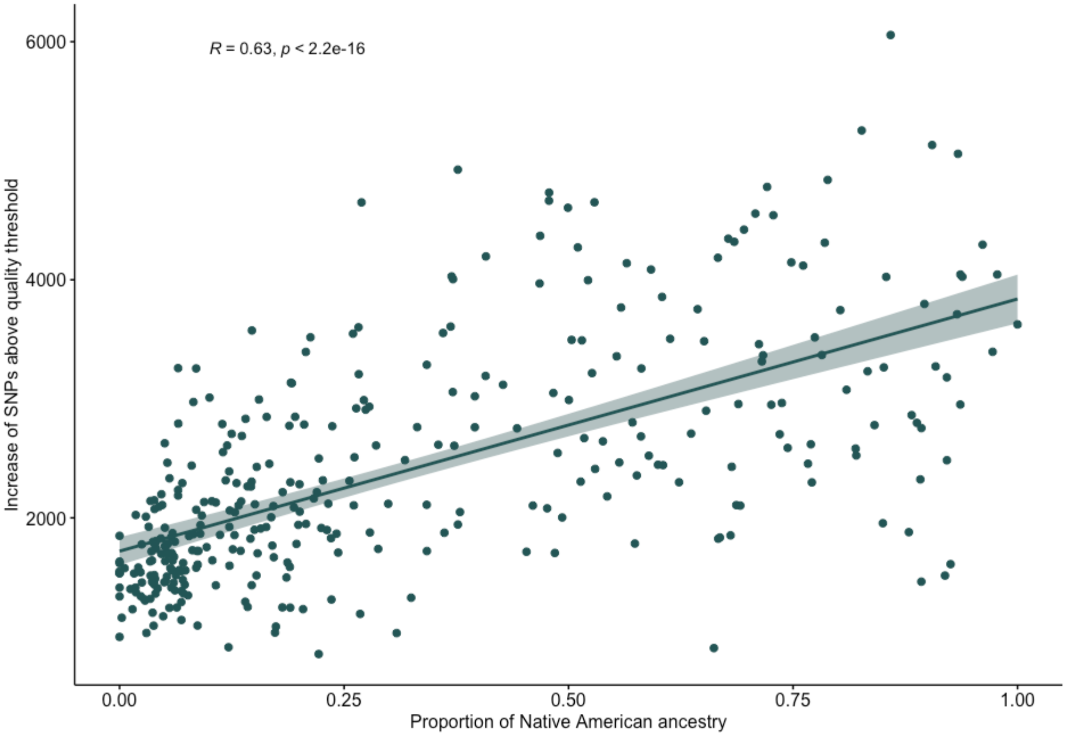

Supplementary figure 1. Correlation between Native American ancestry proportion and the increase of SNPs above the quality threshold using the NATS reference panel in AMR individuals. The increase of SNPs is defined by the difference between the number of SNPs above the quality threshold using 1KGP + NATS and 1KGP alone. We show the Pearson correlation coefficient and the p-value calculated using a two tailed t-test.

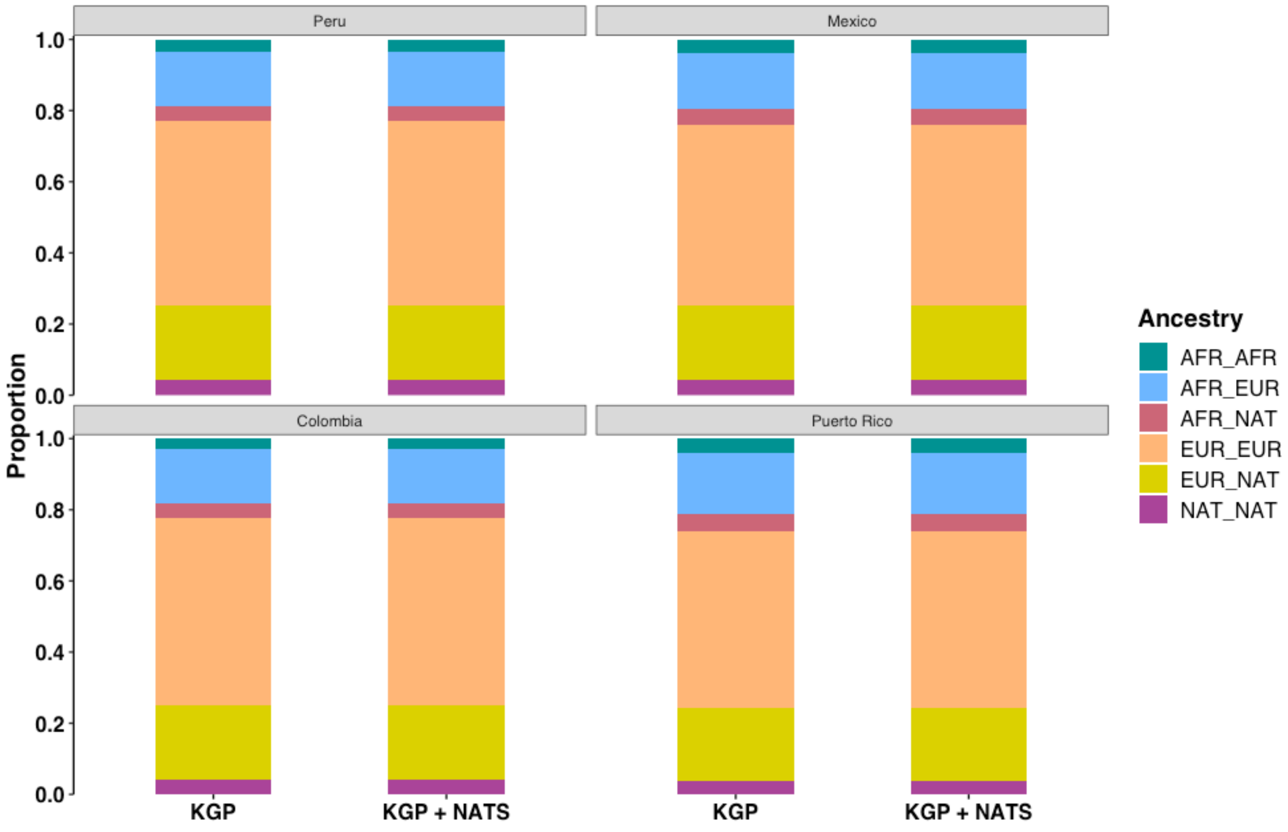

Supplementary figure 2. Proportion of SNPs above the quality threshold by diploid ancestry after imputation using 1KGP and 1KPG + NATS as reference panels. Colors represent the possible diploid ancestries.

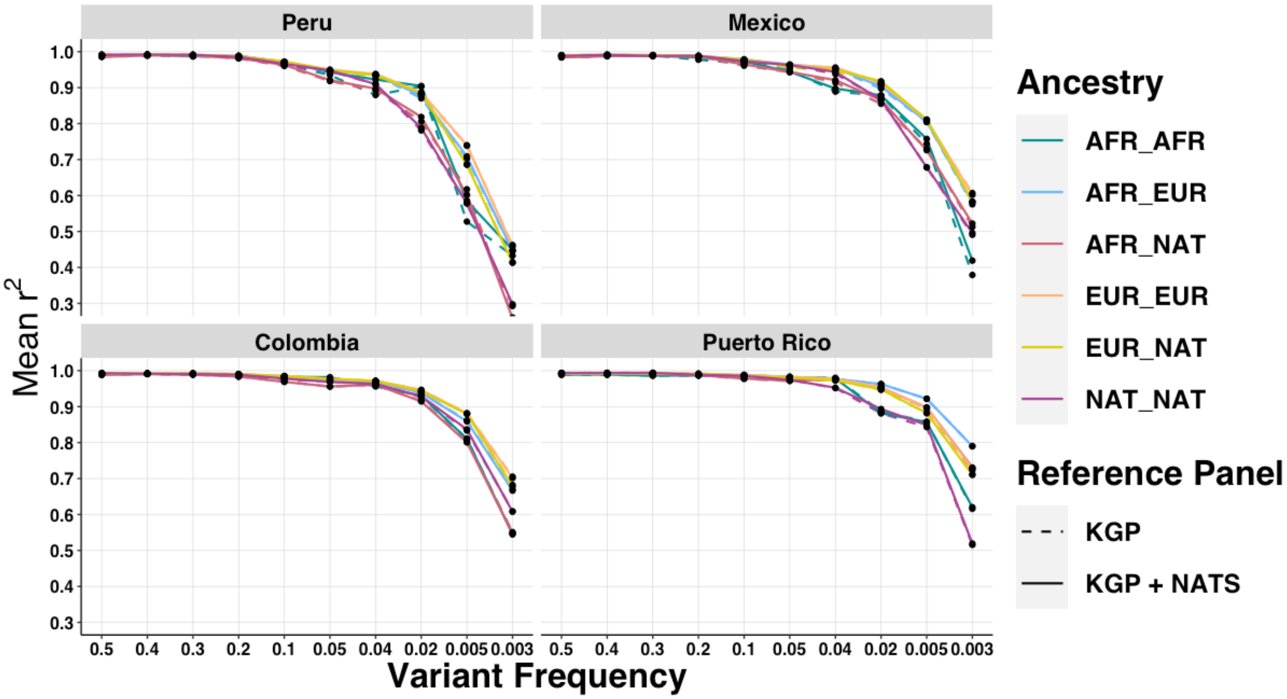

Supplementary figure 3. Imputation accuracy for all ancestries using 1KGP and 1KGP + NATS as reference panel. Imputation accuracy was calculated with the Pearson squared correlation between imputed and real allele dosages.

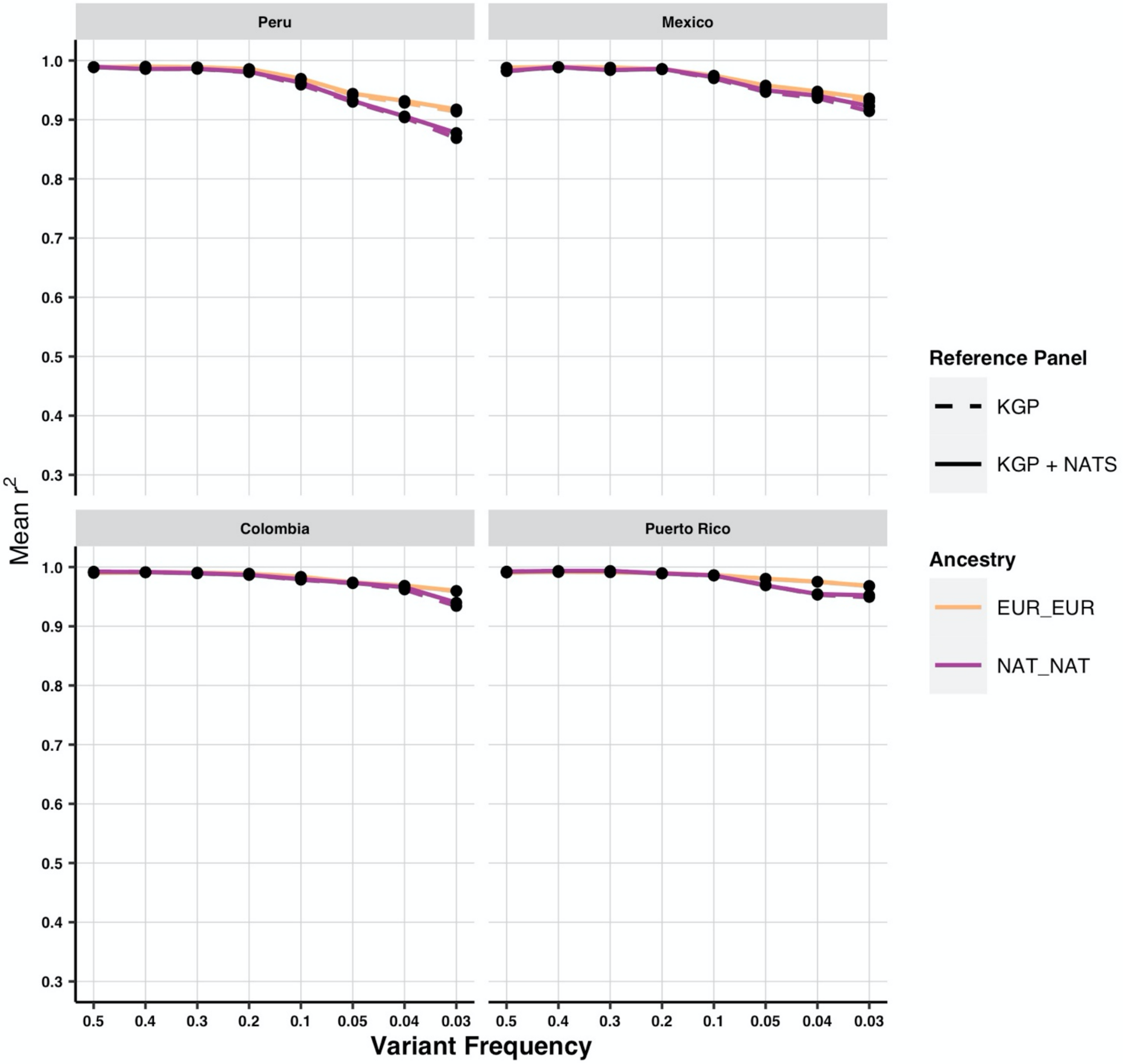

Supplementary figure 4. Imputation accuracy for common variants with NAT and EUR ancestry using KGP and KGP + NATS as reference panel. Imputation accuracy was calculated with Pearson squared correlation between imputed and real allele dosages.

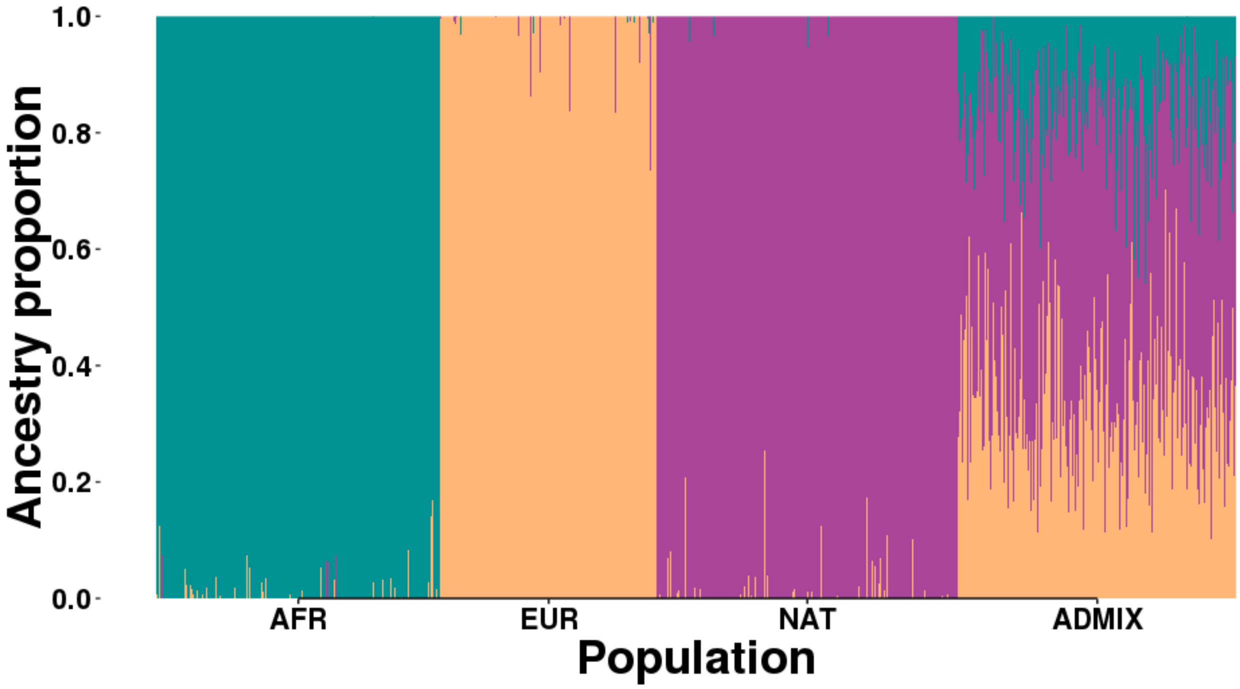

Supplementary figure 5. Admixture plot K = 3 of the simulated data set. Ancestry proportions were calculated with ADMIXTURE. To reduce computing time, we subsetted 700 Native American individuals from the original 3000. Given the available demographic model (Browning et al., 2018), a simulated Asian population was used as a proxy for Native American ancestry for the purpose of reproducing a 3-way admixture process similar to that experienced by admixed Latino populations (ADMIX), which derive from African (AFR), European (EUR), and Native American (NAT) ancestry sources (see Methods for details).

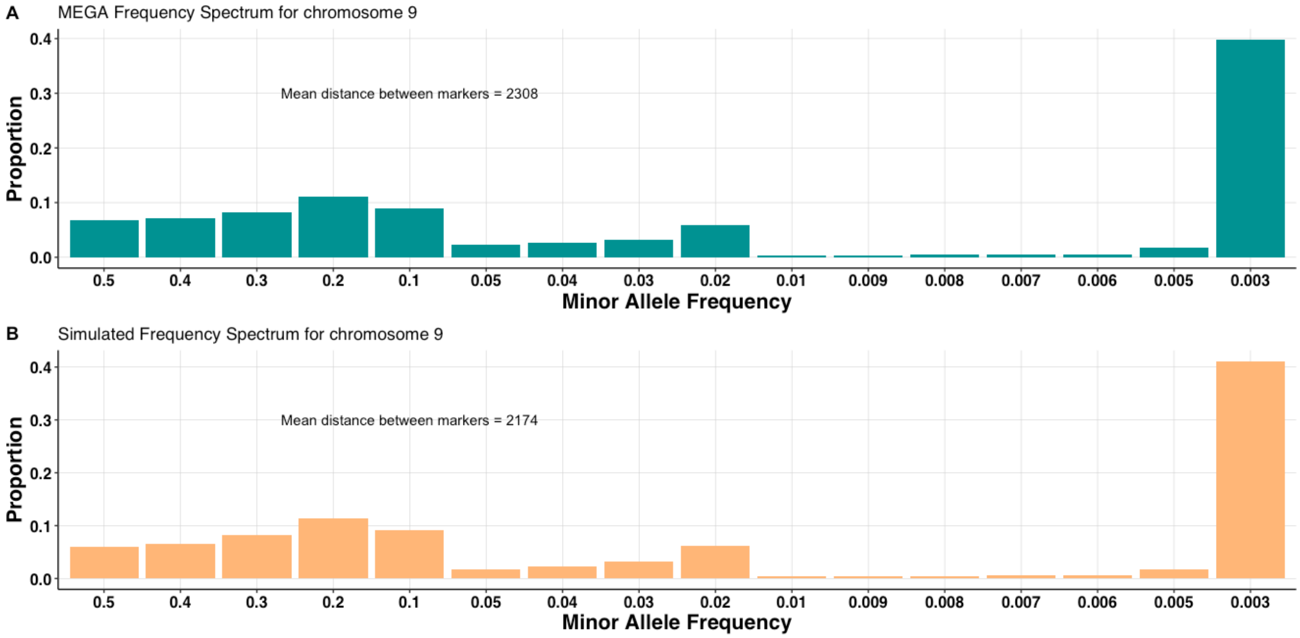

Supplementary figure 6. Proportion of SNPs by Minor Allele Frequency bin of arrays. A) Proportion of SNPs by Minor Allele Frequency bin and mean distance between SNPs of the MEGA array in the EUR population of 1KGP. B) Proportion of SNPs by Minor Allele Frequency bin and mean distance between SNPs of the simulated array in the EUR population of the simulation.

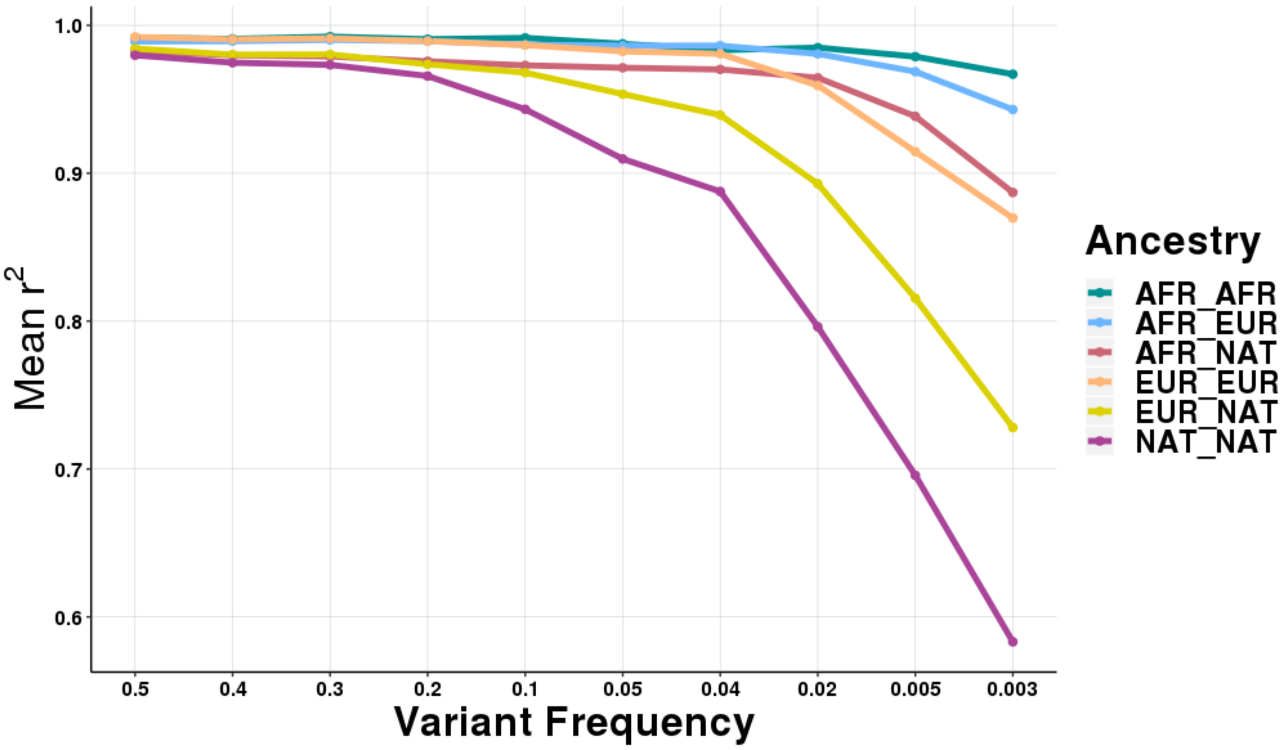

Supplementary figure 7. Imputation accuracy of the simulated base reference panel by diploid ancestry. Imputation accuracy of the simulated base reference panel (0 natives). Imputation accuracy was calculated with the Pearson squared correlation between imputed and real allele dosages.

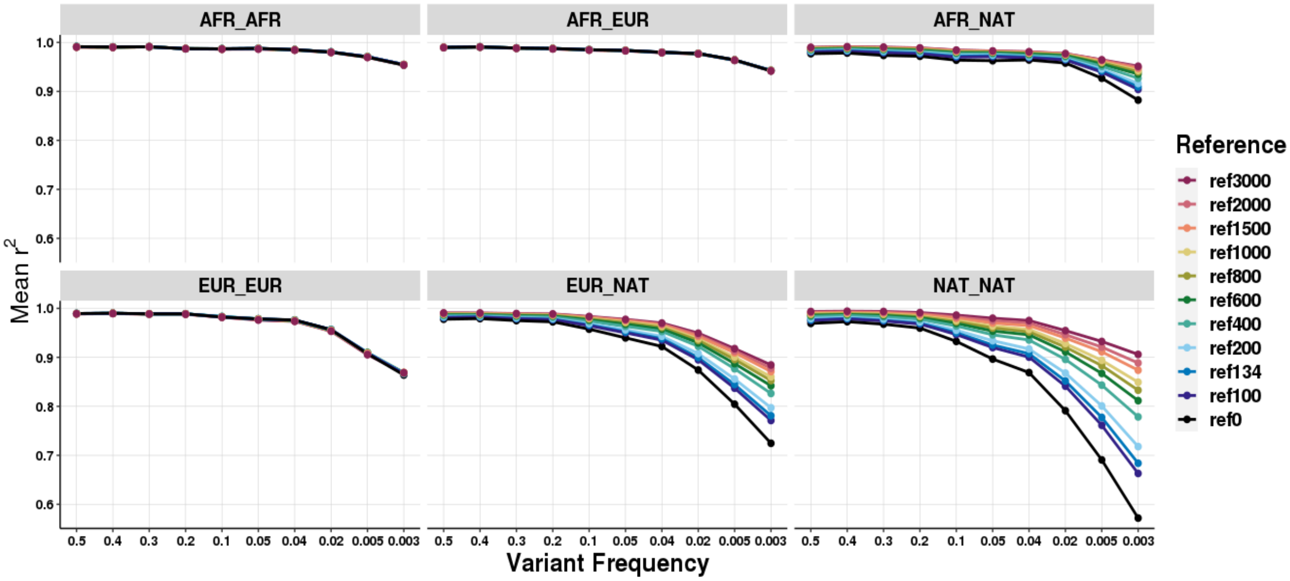

Supplementary figure 8. Imputation accuracy of the simulated reference panels by diploid ancestry. Each panel shows a different diploid ancestry. Colors represent the different reference panels of incremental sizes. Ref 0 stands for the base reference (as it has 0 additional reference genomes). Given the available demographic model (Browning et al. 2018), a simulated Asian population was used as a proxy for Native American ancestry for the purpose of reproducing a 3-way admixture process with similar ancestry proportions of African, European, and Native American sources to that observed in admixed Latino populations (see Methods for details). Imputation accuracy was calculated with the Pearson squared correlation between imputed and real allele dosages.

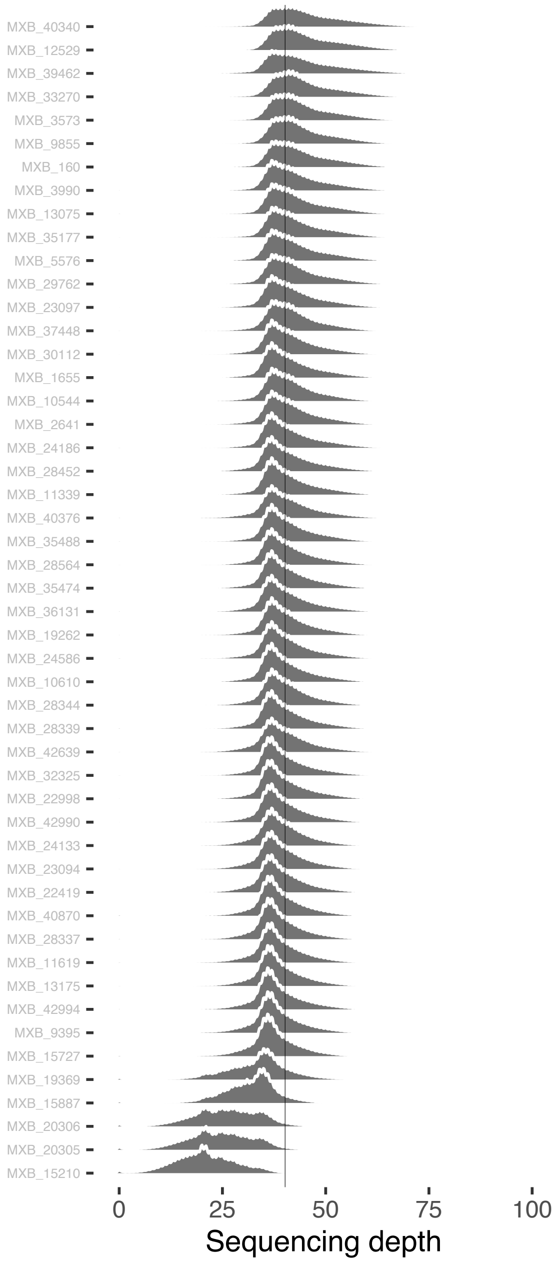

Supplementary figure 9. Sequencing depth for the 50 newly sequenced genomes from the MX Biobank Project. The distribution of read coverage for a random fraction of variants (10%) is shown for each sample. Data points with depth > 100 reads were removed for visualization purposes. The vertical line indicates the global average and individual averages are available in Supplementary Table 2.
